# Supplementary material for: Identification of therapeutics that target eEF1A2 and upregulate utrophin A translation in dystrophic muscles
Source: Nat Commun. 2020 Apr 24;11:1990. doi: 10.1038/s41467-020-15971-w (PMC7181625; doi:10.1038/s41467-020-15971-w)
Supplement: Supplementary file 1 — Supplementary Information [file 41467_2020_15971_MOESM1_ESM.pdf]

Supplementary Information

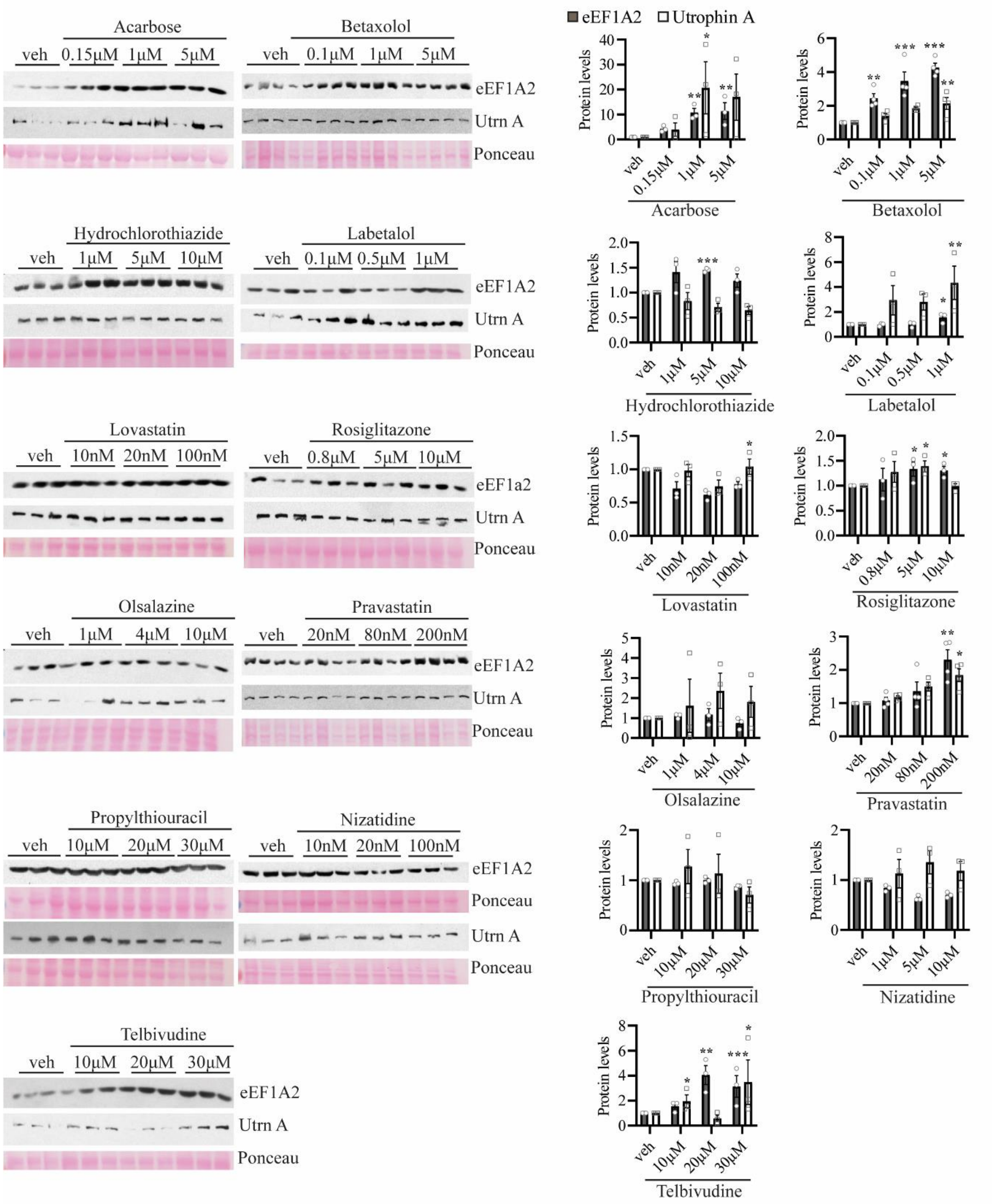

Supplementary Figure 1. Confirmation of eEF1A2 and utrophin A protein expression level increases in C2C12 cells, post FDA-approved drug treatment. Myoblasts were treated with 3 different concentrations of the 11 eEF1A2-activating drugs identified from the screen, including the concentration used in the high throughput screen, for 24 hours. Protein extracts were subjected to western blot analysis. Western blots and quantifications of eEF1A2 and utrophin A protein

levels normalized to ponceau. Table 2 summarizes the fold increase of both proteins to vehicle control (N=3). Error bars represent SEM, *P < 0.05, **P < 0.01, ***P < 0.001, significantly different from vehicle control. Source data are provided as a Source Data file.

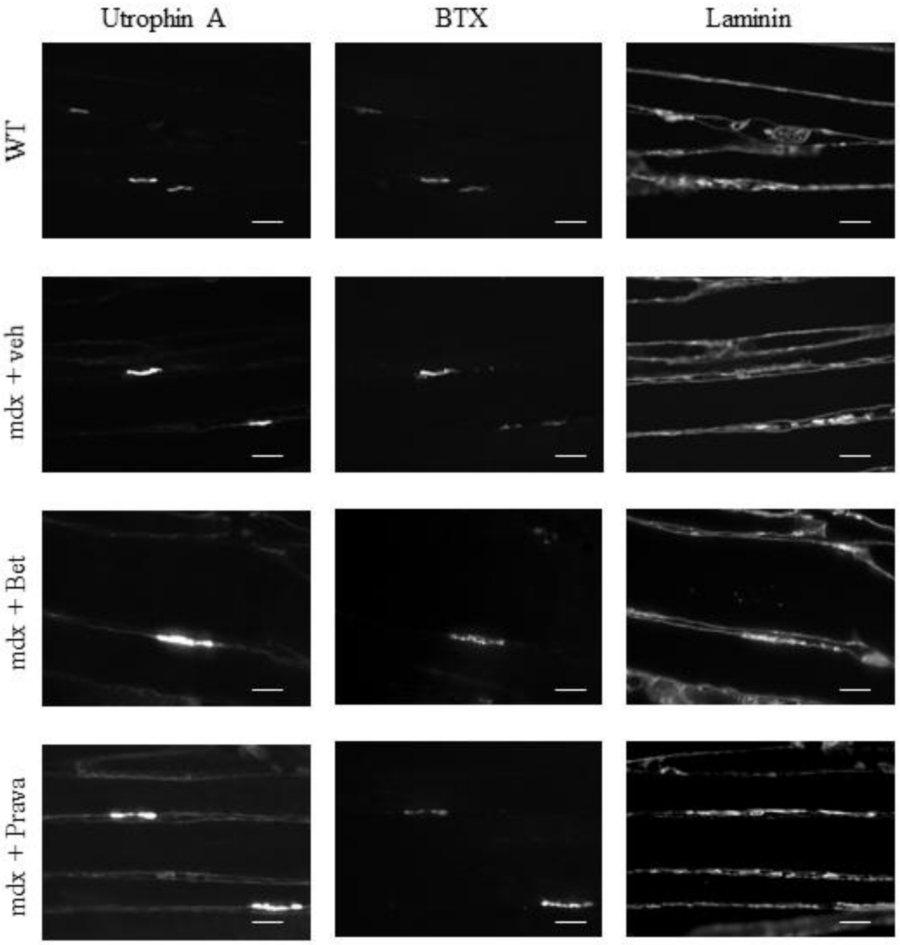

Supplementary Figure 2. Utrophin A localization at NMJ and into extrasynaptic regions of muscle fibers in Betaxolol- and Pravastatin- treated mdx mouse muscles. A) Representative examples of longitudinal sections of TA muscles of wild-type (WT) mice and mdx mice treated with Betaxolol (Bet), Pravastatin (Prava) or vehicle control (saline), were stained for utrophin A, Bungarotoxin (BTX) highlighting the NMJ, and laminin. Scale bars, 50 mm. (N=5).

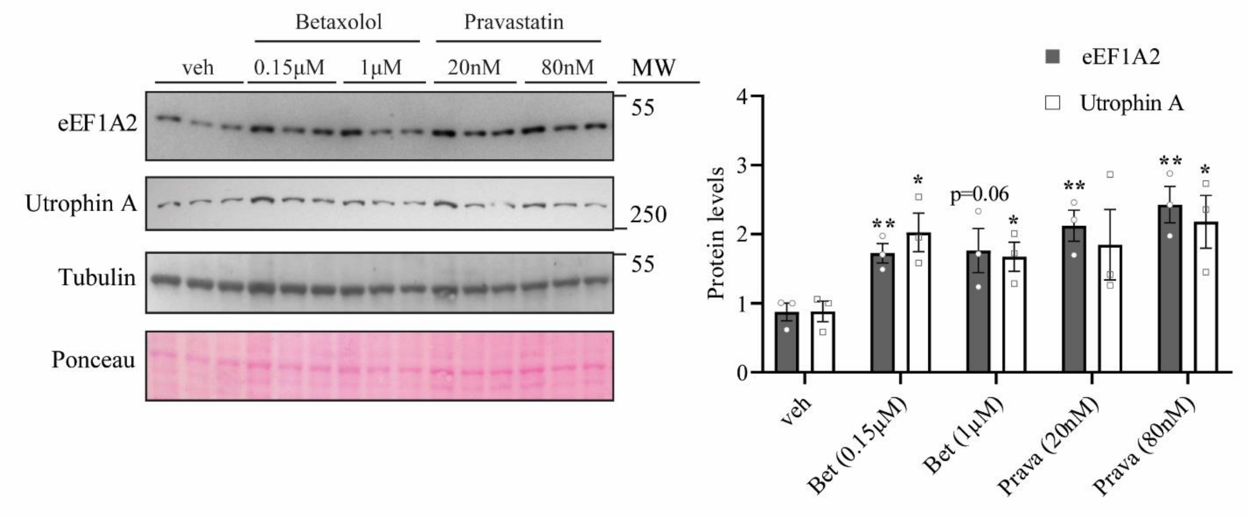

Supplementary Figure 3. Betaxolol and Pravastatin increase eEF1A2 and utrophin A protein expression levels in human skeletal muscle cells. Human skeletal muscle cells (SkMC) were treated with either Betaxolol or Pravastatin for 24 hours. Protein extracts were subjected to western blot analysis. Western blots and quantifications of eEF1A2 and utrophin A protein levels were normalized to tubulin. (N=3). Error bars represent SEM, *P < 0.05, **P < 0.01, significantly different from vehicle control. Source data are provided as a Source Data file.

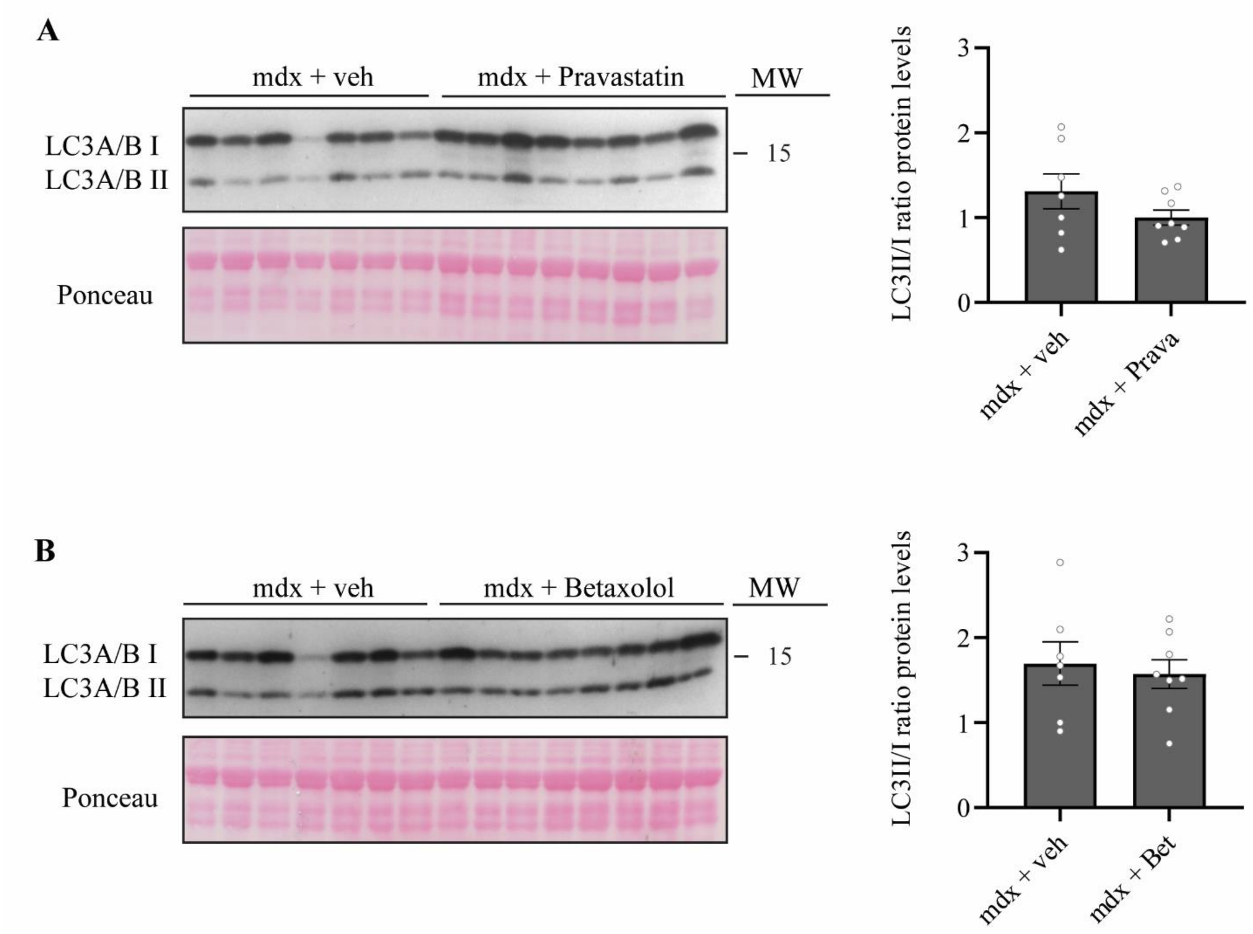

Supplementary Figure 4. Pravastatin and Betaxolol have no effect on autophagy in mdx mice muscle. Lysates obtained from TA muscles of wild-type (WT) mice and mdx mice treated with Betaxolol (Bet), Pravastatin (Prava) or vehicle control (saline) for 4-weeks were subjected to western blot analysis. To determine the effect of our drug treatments on autophagy, we probed for autophagy markers LC3A/B I and II. An increase in the ratios LC3II to LC3I would be representative of an increase in autophagy, however there are no significant differences between control and treated groups. Quantifications of LC3I and II protein levels was normalized to ponceau. (N=8). Error bars represent SEM. Source data are provided as a Source Data file.
